# Work Engagement in Nurses during the Covid-19 Pandemic: A Cross-Sectional Study

**DOI:** 10.3390/healthcare9030253

**Published:** 2021-03-01

**Authors:** Regina Allande-Cussó, Juan Jesús García-Iglesias, Carlos Ruiz-Frutos, Sara Domínguez-Salas, Carmen Rodríguez-Domínguez, Juan Gómez-Salgado

**Affiliations:** 1 Nursing Department, Nursing, Physiotherapy and Podiatry School, University of Seville, 41009 Seville, Spain; 2 Sociology, Social Work, and Public Health Department, University of Huelva, 21007 Huelva, Spain; juanjesus.garcia@dstso.uhu.es (J.J.G.-I.); frutos@dbasp.uhu.es (C.R.-F.); salgado@uhu.es (J.G.-S.); 3 Postgraduate Programme on Safety and Health, Universidad Espíritu Santo, Samborondón 092301, Ecuador; 4 Psychology Department, Universidad Loyola Andalucía, Avda. De las Universidades s/n, 41704 Dos Hermanas, Spain; sdominguez@uloyola.es (S.D.-S.); mcrodriguez@uloyola.es (C.R.-D.)

**Keywords:** work engagement, nurses, COVID-19, mental health, assessment studies

## Abstract

In some areas of Spain, health services and professionals working in the front line against the Sars-Cov-2 virus have been widely overwhelmed at all levels. Therefore, the objective of this study was to assess the level of work engagement of Spanish nurses during the COVID-19 pandemic. A cross-sectional study was carried out. The sample consisted of 510 active nurses from all over Spain, without age exclusion, who voluntarily accepted to participate in the study. Work engagement was assessed with the 9-item Utrecht Work Engagement Scale (UWES) questionnaire, through an online questionnaire and non-probabilistic snowball sampling. The results showed a mean age of 45.9 years (SD = 10.7 years), most of them women (78.1%), and 58.5% were in primary care. The mean score for the UWES-9 questionnaire was 4.6 points (SD = 1.35). The categorical regression analysis performed revealed an R^2^ value of 0.75 and a significance of *p* < 0.01 in the sex, type of unit, and training variables. The Spanish nurses in the sample present high levels of work engagement in all dimensions in general, although the lowest mean scores are found in the vigor dimension, among men, and nurses working in hospital and critical units.

## 1. Introduction

The pandemic caused by the Severe Acute Respiratory Syndrome coronavirus 2 (SARS-CoV-2) is having dire social, cultural, and economic consequences, to a greater or lesser extent, in all countries worldwide [1]. The disease that causes SARS-CoV-2 was named by the World Health Organization (WHO) as COVID-19 and can present a very variable symptomatology: from asymptomatic individuals, to others who have symptoms of mild severity such as fever, dry cough, tiredness, anosmia, dysgeusia, etc [2]. In the most severe cases, it can trigger severe respiratory failure syndrome, with multiorgan failure, as a result of the so-called “cytokine storm” that can lead to the death of the individual [3]. In this sense, since the beginning of the pandemic, the Johns Hopkins Coronavirus Resource Centre has reported to date 1,125,752 deaths worldwide [4] and, in the specific case of Spain, 33,992 deaths and 281,388 active cases. An incidence rate is above 3000 cases in autonomous communities of the north and center of Spain (Madrid, Navarra, Aragon, and Castilla León) [5].

To counter this situation, governments in all countries have taken steps to reduce the onslaught of the disease and minimize the health, economic, and social consequences [1]. In countries such as Spain, since the entry into force of the Royal Decree 463/2020 of 14 March declaring the state of alarm for the management of the health crisis situation caused by COVID-19 [6,7], a number of measures have been taken as the outbreak progressed. Some of these measures were of social distancing, measures to limit the mobility of people (confinements, temporary abolition of the right to free movement of persons and vehicles, etc.) [8], proposals for hygienic measures (hand washing, respiratory hygiene, mask use, among others), and contact history search and tracking. The sole purpose was of preventing the spread of the virus and avoiding the overload of care services [1,9]. Despite this, in some regions of Spain, especially in large cities, health services and professionals working in the front line against the virus, both health workers (doctors, nurses, among others) and non-health staff (wardens, cleaning personnel, security personnel, and state forces, among others) have been widely overwhelmed at all levels [10]. From an organizational point of view, it has been necessary to restructure all care units to cope with the disease (Covid-19 floors, adapted Critical Care Units, cleaning itinerary, and Covid-19 itinerary for patient entry and staff relocation, etc.) [11] In addition, the lack of material resources for patients such as respirators and nasal goggles and personal protective equipment for professionals such as gloves, gowns, and masks, being in some cases reused [12], both in health facilities (hospitals and health centers) and in social health centers (elderly homes), should be taken into account. In this sense, the lack of material and the close contact established during the treatment of patients infected with SARS-CoV-2 leads to healthcare professionals having an increased risk of being infected. This is especially the case among nurses, as they are the collective that spends the most time and performs the most interventions on patients [13,14]. In Spain, a total of 86,000 health workers have been infected and 63 have died from the disease as of November 20, accounting for approximately 5.28% of all the people infected [5].

Being a nurse in COVID-19 times can be a difficult profession, and is not free of risk. In addition to what was previously exposed (lack of protection, risk of contagion, and restructuring of services), the mental health of nurses in the performance of their duties has been harmed [14] and symptoms related to stress, depression, anxiety, insomnia, etc., have manifested [15]. Throughout the long working days, nurses have faced difficult ethical and moral judgments, as they have accompanied patients in solitude and said goodbye to them in their final hours [11]. In addition, they have worked long hours equipped with complete personal protective equipment, which has sometimes caused pressure injuries, and made them suffer physical exhaustion, fear, and concern for infecting their family members after work [16,17]. Although much of society considers nurses as heroines, there are also small trouble spots where nurses have suffered stigmatization and feelings of isolation as being considered possible “carriers of the disease” [17].

Despite all these negative conditions, nurses have continued providing service. This fact can be explained by the individual attributes of nurses that are used to develop some resilience [18], resistance, vigor, and self-efficacy; they are possible protective factors for the mental health of nurses in the face of an adverse situation such as a health crisis [19]. At the organizational level, perceiving support from society, having confidence in the team, and feeling a useful and necessary part of care (sense of belonging) [18,20] can reduce stress and exhaustion and improve the mental health of nurses. Owning protective equipment, receiving training, and psychological and emotional support, as well as showing greater commitment to their work [19] can also reduce their stress levels. In order to protect the mental health of health professionals from the impact of the pandemic, some authors have proposed the need for institutions to implement interventions aimed at creating a psychologically safe environment. Moreover, a strong leadership, clear organizational strategies for staff well-being, constant communication, and meaningful team support have been identified as protective interventions [21]. One of the focuses of intervention for the improvement of well-being is engagement, a way to avoid the prevalence of stress or burnout among health workers. It is considered as a positive organizational psychology perspective [22] and an indicator of intrinsic motivation for work [23]. Some authors suggest that work engagement is a protective factor against exhaustion [24] and is understood as “a positive, satisfactory and work-related mental state characterised by vigour, dedication, and absorption” [25]. It is therefore a construct composed of three dimensions: physical (vigor), emotional (dedication), and cognitive (absorption). The first dimension, vigor, refers to the level of energy, will of effort, perseverance, and mental resilience that a person has in work, even when facing some difficulty. The second one, dedication, refers to how involved a person is with work at the level of enthusiasm, inspiration, pride, and challenge. Finally, absorption refers to the degree of concentration and abstraction a person has when working [25].

Prior to the health emergency caused by COVID-19, some studies in Spain have estimated that the level of work engagement among nurses was categorized as moderate, with higher scores in the vigor and dedication dimensions and lower in the absorption dimension [26,27]. However, some variables such as the unit in which the nursing professional works and the pressure to provide care, which can influence the level of work engagement [28], were not studied in depth. Therefore, the objective of this study was to assess the work engagement of Spanish nurses during the first wave of the COVID-19 pandemic, considering sociodemographic and work-related variables.

## 2. Materials and Methods

### 2.1. Design

A descriptive cross-sectional study was carried out according to the classification by Herzog et al. [29] during the months from March to June 2020.

### 2.2. Population and Sample

A sample of at least 192 subjects was estimated, considering that the sampling population was 255,473 active nurses in Spain [30], with a confidence level of 95%, an accuracy of 2.5%, and an expected proportion of losses of 25%, and a statistical power of 80%. However, the final sample was composed of 510 active nurses from all over Spain, increasing the statistical power to 95%. The sample was composed of subjects without age exclusion, who voluntarily accepted their participation in the study.

The sample was selected by non-probabilistic snowball sampling. An online questionnaire was created with a Google Forms application (Google, City of Mountain View, CA, USA), which contained information about the study and reported knowledge, as well as other items related to the study variables. The link to the questionnaire was disseminated by e-mail to official nursing groups, identified with the help of the General Council of Nursing of Spain. The management of the different groups disseminated it among their associates, who had agreed to receive emails for research purposes. All subjects were informed of the purpose of the study and the possibility of voluntarily, anonymously, and confidentially participating in it once they accessed the questionnaire link. In addition, they all had to give their consent to participate by marking a specific box. Following this action, the subject could access the questionnaire questions; otherwise, these could not be accessed.

### 2.3. Variables

This study included sociodemographic variables (sex, age, province of work, maximum level of training, research activity, and teaching activity), work-related variables (years of experience, level of care in which they serve, and type of unit), and those related to the measuring instrument (vigor, dedication, and absorption).

### 2.4. Instrument

Work engagement was assessed using the Utrecht Work Engagement Scale (UWES) 9-item questionnaire, with a Likert-type response scale between 0 (never) and 6 (always) [25]. Work engagement is a construct composed of three dimensions: physical (vigor), emotional (dedication), and cognitive (absorption). The first dimension, vigor, refers to the level of energy, will of effort, perseverance, and mental resilience that a person has in work, even when facing some difficulty. The second one, dedication, refers to how involved a person is with work at the level of enthusiasm, inspiration, pride, and challenge. Finally, absorption refers to the degree of concentration and abstraction a person has when working [25]. Its Spanish validation obtained a Cronbach’s alpha value of 0.9, and between 0.79 and 0.84 for the three subscales [25].

The values of the means scores of UWES are: very low 0 to 0.99; low 1 to 1.99; medium 2 to 3.99; high 4 to 4.99; very high 5 to 6.

### 2.5. Data Analysis

Univariate and bivariate descriptive data analysis was performed using the IBM SPSS Software (IBM, Armonk, NY, USA). The Kolmogorov–Smirnov test demonstrated the lack of normality in data distribution, so nonparametric statistics were used. Descriptive central trend and dispersion statistics were performed for data analysis, as well as correlations and contrast tests such as Mann–Whitney U, Kruskal–Wallis, Kendall Tau B, or Spearman’s Rho.

In order to establish the relationship between work engagement (total score of the UWES-9 questionnaire) and the other variables, the categorical regression analysis (CATREG) was performed since these are qualitative in nature. The CATREG analysis includes characteristic aspects of classic regression analysis: coefficient of determination (R2), variance analysis in regression, and significance of the model’s parameters. For calculation, the optimal scaling option in the SPSS© software was selected.

### 2.6. Ethical Aspects

Due permission was obtained from the Research Ethics Committee of the regional Government of Andalusia (Ref. 1539-N-20), as well as from the General Council of Nursing of Spain. In addition, all subjects of the sample voluntarily signed an informed consent, having been informed of the purpose of the study and ensuring at all times the confidentiality of the data and the anonymity of the participants, based on Organic Law 3/2018 of December 5 for the protection of personal data and the guarantee of digital rights.

If the participant did not check off the box for informed consent, it was not possible for them to access the questionnaire.

## 3. Results

The results showed a mean age of 45.9 years (SD = 10.7 years), most of the participants being women (77.4%) (Table 1). Of the total sample, 45% had obtained a university diploma in nursing, 15.3% a degree in nursing, and 23.2% held a Master’s degree; 80.3% of the sample did not carry out any research activity, and 51.7% were not teaching. In addition, 59% were in hospital care, and the mean number of years of professional experience was 22.3 years (SD = 11.1 years) (Table 1).

Of the 50 provinces of Spain, Seville (*n* = 195), Madrid (*n* = 70), and Barcelona (*n* = 46) gave the highest frequencies. The provinces of Cuenca, Albacete, Girona, Palencia, and Segovia had no representation. The remaining 42 provinces, together with the autonomous cities of Ceuta and Melilla, obtained the least frequencies from two subjects.

Regarding the score obtained in the UWES-9 questionnaire, the mean was 4.6 points (SD = 1.35) (Table 1). In addition, in the distribution of mean scores, 80% of the sample showed intermediate and high levels of work engagement, the mode being the “high level” category (180 subjects; 33% of the sample). The “vigour” dimension scored the lowest mean, with 4.46 points (SD) = 1.44). On the other hand, the “dedication” dimension achieved a mean score of 4.67 (SD = 1.50), and the “absorption” dimension, 4.55 (SD = 1.50). However, for all three dimensions, 80% of professionals showed intermediate, high, and very high levels, with the category “high” being the mode in the three subscales.

On the other hand, the Kolmogorov–Smirnov test, with a value of *p* < 0.01, revealed that the distribution of scores did not follow normality. With regard to the bivariate analysis, no significant differences were found in the mean questionnaire score for the research activity variables (H Kruskal–Wallis *p* = 0.19) or for the teaching activity variable (H Kruskal–Wallis *p* = 0.52). In contrast, there are significant differences for the sex (Mann–Whitney U *p* < 0.01), training (H Kruska–Wallis *p* < 0.01), level of care (H Kruskal–Wallis *p* = 0.01), and type of unit (H Kruskal–Wallis *p* = 0.049) variables. Thus, the group of women (no. 395) obtained a mean score of 4.70 points, while for the group of men it was 4.30 (No. 115). In addition, professionals in possession of a University Specialist degree scored a mean of 5 points (No. 76), while for the rest of the degrees this stood between 4.5 and 4.7. In addition, nursing professionals who were active in Primary Care (No. 176) and Mental Health (No. 13) offered mean scores of 4.87 and 4.81, respectively. Professionals linked to hospital medical units (No. 76) and hospital surgical units (No. 66) had the lowest mean scores of 4.42 and 4.43, respectively.

There was no significant correlation between age and total mean UWES-9 score, so both variables are not associated (Tau b = 0.004). In the same way, there were no significant correlations between mean scores for the three subscales of the UWES-9 questionnaire and total mean UWES-9 scores (Tau b rated between 0.01 to 0.3).

Table 2 presents the distribution of mean scores for each dimension regarding the values of the sex, level of care, training, and type of unit variables.

The results show that the lowest mean scores are found in the vigor dimension, in the group of men and in nursing professionals working in hospital medical units (4.19 points in both cases). In contrast, the highest mean score is present in the abstraction dimension (5.16 points) by those subjects who alternately work in both primary care and specialized care due to recruitment as temporary staff. Likewise, with a score of 5.12 points, these data are followed by those subjects who hold a degree in nursing as the highest academic degree, for the dimension of dedication. The remaining score ranges from 4.20 to 5 points, depending on the values of each variable and dimension of the UWES-9 questionnaire.

The categorical regression analysis performed, with the total score of the UWES-9 questionnaire as a dependent variable and the other variables, revealed an R^2^ value of 0.75 and a significance of *p* < 0.01 (see Table 3). In addition, the individual significance of the sex, type of unit, and training variables in the model revealed *p*-values < 0.05. However, the level of care variable was not significant in the tested model, with a value of *p* > 0.05.

Categorical regressions were also performed by setting as a dependent variable the mean score for the three subscales of the UWES-9 questionnaire. In the case of vigor and dedication, the significant variables in the model were sex, type of unit, and training, as in the model tested with the total mean UWES-9 score (see Table 3). The model adjustment also got an R^2^ value of 0.75, and the *p*-values obtained by the three variables were <0.01. However, when the dependent variable was the mean score in the abstraction subscale, the model also significantly included the level of care variable, with a value of *p* = 0.02, keeping all other variables also significant.

## 4. Discussion

In view of the impact of the current pandemic, the objective of this study was to evaluate the level of work engagement of Spanish nurses during the early stages of the health crisis caused by COVID-19 disease in Spain, considering sociodemographic and work-related variables. The results showed a mean age of 45.9 years (SD = 10.7 years), most of the participants being women (77.4%). Of the total sample, 45% had obtained a university diploma in nursing and 15.3% a degree in nursing. In addition, 59% were in hospital care. These findings are in line with the data published by the National Institute of Statistics in Spain in 2019. The mean age was 44 years old, and 80% of Spanish nurses are women. Most of them achieved a university diploma in nursing, because the new degree in nursing was implemented in Spain in 2010. Moreover, the Spanish National Health Service describes that 60% of the Spanish nurses work in hospital wards, and 40% in primary cares and other type of settings [30].

The mean work engagement score obtained in this study was 4.6 points (SD = 1.35). This needs to be compared with scores obtained in studies prior to the COVID-19 pandemic. Thus, a study in 2019 on 527 nurses in southern Spain yielded total work engagement results of 4.00 (SD = 1.2) [27]. In another study carried out in 2011 on 508 nurses from four general hospitals in the community of Madrid (the center of the country), the score obtained for work engagement rose to 4.10 (SD = 0.6) [31]. Another study carried out in the northeast geographical area of Spain described work engagement levels of 4.49 (SD = 0.86) [26]. In this context, in addition to geographical differences, it appears that during the COVID-19 pandemic there could be a slightly higher score on work engagement, as compared to the values obtained in previous studies. In this sense, it should be clarified that when nurses feel committed to work, they experience high levels of energy and are absorbed in work, increasing self-realization, giving more meaning to the work they develop, and engaging themselves in it [27]. Similarly, high levels of work engagement favors nurses who are able to find and mobilize new work resources if necessary, such as asking for help or advice from colleagues and supervisors [28]. Similarly, some authors agree that the media can positively favor how society sees workers, and this would increase the level of self-esteem and their sense of belonging, while also offering a positive image [31]. However, these results differ from Kim et al.’s findings [32] where in their study on a sample of 377 South Korean nurses, no significant differences between social support and COVID-19 pandemic levels of work engagement were found.

Other authors believe that leadership styles and support from superiors in times of health crisis could increase workers’ work engagement [33], and this in turn would help reduce the risk of developing major depressive episodes [34]. Thus, Zhang et al. [35] emphasized that the psychological and labor support from superiors, and the improvement of the perception of autonomy of any employee, could increase work engagement [34], though the authors themselves argue that future research should address this issue in depth.

With regard to work engagement, from an international perspective, studies such as the one by Ahmed et al. [36] on a sample of 497 registered nurses from five hospitals in Wuhan offered even higher values in levels of work engagement during the health crisis. In fact, they obtained high values in vigor 5.39 (SD = 0.46), dedication 5.51 (SD = 0.41), and total work engagement 5.44 (SD = 0.41), and very high values in absorption 5.43 (SD = 0.45). In another study on another sample of 242 nurses from various health areas of Wuhan, China, total work engagement scores were of 4.83 (SD = 1.01) [35]. These authors indicate that work engagement may vary depending on the behavior and the level of commitment of the population. In all the studies described above and in the present one, low levels of vigor have been found. This fact could be explained by the characteristics of the nurses’ work context: long working hours, overload, lack of professional experience, and/or closeness to recurring death situations [24].

The present study, like the one by Guo et al. [37], found significant differences (*p* < 0.05) in the sex, type of unit, and level of training variables for the work engagement dimensions. However, other authors did not report significant variations between sex and levels of dedication, absorption, and vigor [38]. Regarding differences found according to the sex variable, Kim et al.’s study [32] described lower work engagement scores among women. However, the greater presence of female nurses at the international level should be considered, and this is likely to lead to a bias in studies in which sex variables are considered in the field of nursing.

It is especially striking how nursing graduates along with people who have achieved a university specialism offer the highest work engagement scores in all dimensions. In Spain, nursing graduates are professionals whose degree is based on the Bologna Plan, created as a European Area of Higher Education [39], and the first graduated nurses from this plan did so from Spanish universities in 2013 so, a priori, they should have less experience. However, it is possible to discuss whether the new training degree incorporates content related to improving the perception of safety, confidence, motivation, and/or confrontation, which has characterized healthcare in recent times. Likewise, it may be thought that postgraduate training makes it possible to acquire advanced skills, perhaps related to the increased sense of control and mastery of the task, and the possibility of dealing better with situations of high psychological demand. Studies that compare these training programs should therefore be developed with regard to their impact on the confrontation and resilience of nurses in situations of high psychological demand.

There is no doubt that the working climate is a key and determining element of the levels of work engagement among nurses and, as such, there have been differences between each of the services where nurses carry out their work. In fact, our results can be in line with studies such as those by Da Silva et al. [38] and Lourençao et al. [40] in which higher levels of work engagement are observed in primary care nurses as compared to those performing their functions in other types of units. This phenomenon could be justified by the better working conditions to which these nurses are exposed, because in the field of primary care in Spain, nurses work only in the daytime and do not work during weekends.

Recent studies have shown that improving the level of work engagement of nurses increases job satisfaction and the sense of coherence, and therefore also their emotional well-being in the development of their profession [15,36]. This improvement in emotional well-being, closely related to increased work engagement, promotes increased resilience to the stress and work overload that characterizes healthcare needs in the current COVID-19 pandemic [15].

Despite the difficulties of the health crisis, the work engagement levels of the sample nurses were high during the first wave of the pandemic. Nurses working in medical and critical hospital units obtained lower mean scores—46.6% for Spanish nurses—so the Spanish health system should design and implement strategies to increase these scores, to improve the job satisfaction and work engagement and to prevent the intention to leave, in these settings [41]. Moreover, the group of men obtained the lowest mean scores in vigor dimension, but they only 22.6% of the sample. Therefore, findings may be not representative of the genre perspective of work engagement. Nevertheless, the mean scores in work engagement obtained in general were high. This fact also reflects social responsibility regarding population care. It is therefore an indicator of job satisfaction that must be measured throughout the progression of the pandemic, to extract from scientific evidence positive factors that help improve the working climate and emotional adjustment of nurses, as well as those variables that need to be modified. Future research should confirm these results and assess the progression of work engagement during the different phases of the current pandemic in Spain, including the post-pandemic context and the genre perspective. The current health crisis could affect the physical, social, mental, and work environment of nurses in the years to come, and improvement strategies can be designed and implemented based on the evidence obtained.

Among the limitations of this study are those of the design with respect to the use of non-probabilistic snowball sampling. This may be a bias affecting the representativeness of the sample. However, it was attempted to avoid it disseminating links to the study among official nursing groups. Likewise, the participants not only declared voluntariness and consent to participate in the study, but also their employment status as an active nurse. In addition, given the use of self-administered questionnaires, researchers should rely on the veracity of the data offered by the study participants. On the other hand, we consider that the moment of the sampling, the working conditions of each subject, and the unequal impact of COVID-19 in Spain have been able to influence the subjects’ responses. In this way, other variables related to the work environment, like team size or availability of equipment, have not been assessed in this study, and should be related to the work engagement levels. Further research is needed in order to study the relationship between both constructs among nurses.

## 5. Conclusions

Spanish nurses in the sample have high levels of work engagement in all dimensions (vigor, absorption, dedication, and total work engagement), being somewhat higher than in studies developed before the COVID-19 pandemic. In addition, significant differences have been found between work engagement levels and sex, type of unit, and education variables (*p* < 0.05): there are higher levels of work engagement in women, in the field of primary care and mental health, and with a nursing training degree and/or a university specialist degree.

## Figures and Tables

**Table 1:** Description of the sample profile.

Variables	Variable Valor	Results	Rank
Age	Mean	40.9	Minimum = 22 Maximum = 68
	Standard Deviation	10.7	
Years of experience	Mean	11.1	Minimum = 1 Maximum = 44
	Standard Deviation	1.5	
**Variables**	**Variable valor**	**Frequency (percentage)**	**Total sample**
Sex	Male	*n* = 115 (22.6%)	Total sample *N* = 510
	Female	*n* = 395 (77.4%)	
Level of training	Nursing Diploma	*n* = 230 (45%)	
	Nursing Degree	*n* = 78 (15.3%)	
	University Master’s	*n* = 100 (20.7%)	
	Specialist	*n* = 76 (14.9%)	
	Doctorate	*n* = 25 (4%)	
Research activity	Yes	*n* = 100 (19.7%)	
	No	*n* = 410 (80.3%)	
Teaching activity	No	*n* = 264 (51.7%)	
	Yes: Clinical Tutor	*n* = 168 (32.9%)	
	Yes: Associate Professor	*n* = 75 (14.8%)	
	Yes: Associate Doctor Prof.	*n* = 3 (0.5%)	
Type of unit	Primary Care	*n* = 187 (36.6%)	
	Hospital: Medical Units	*n* = 83 (16.2%)	
	Hospital: Surgical Units	*n* = 75 (14.8%)	
	Critical Care and Emergencies	*n* = 80 (15.6%)	
	Out-patient Emergencies	*n* = 18 (3.5%)	
	Mental Health	*n* = 13 (2.5%)	
	Mother and Baby Unit	*n* = 24 (4.7%)	
	Patient Management	*n* = 30 (5.8%)	
Uwes-9 score	Mean	4.6	Maximum score = 6 points
	Standard Deviation	1.5	

**Table 2:** Mean scores distribution by UWES-9 dimension and statistical significance.

Variables		*p*-Value	Vigour Mean (SD)	Dedication Mean (SD)	Absorption Mean (SD)	Overall Score Mean (SD)
Sex	Male	0.001	4.19 (1.41) ^a^	4.31 (1.52) ^a^	4.35 (1.38) ^b^	4.30 (1.43) ^a^
	Female		4.54 (1.44) ^a^	4.77 (1.48) ^b^	4.61 (1.63) ^b^	4.70 (1.51) ^b^
Level of care	Specialized Care (SC)	0.001	4.32 (1.44) ^a^	4.55 (1.5) ^a^	4.42 (1.5) ^b^	4.51 (1.51) ^a^
	Primary Care (PC)		4.66 (1.40) ^a^	4.81 (1.52) ^b^	4.70 (1.35) ^b^	4.72 (1.37) ^b^
	SP and PC (alternate periods, according to contract)		4.74 (1.3) ^a^	5.04 (1.51) ^b^	5.16 (1.6) ^b^	4.95 (1.46)
Type of unit	Out-patient Care	0.05	4.65 (1.29) ^a^	4.81 (1.27) ^b^	4.67 (1.29) ^b^	4.69 (1.28)
	Primary Care		4.80 (1.52) ^a^	4.97 (1.56) ^b^	4.87 (1.29) ^b^	4.84 (1.45)
	Critical Care and/or Emergencies		4.23 (1.7) ^a^	4.56 (1.52) ^a^	4.28 (1.69) ^b^	4.44 (1.63) ^a^
	Hospital: Medical Units		4.19 (1.6) ^a^	4.44 (1.57) ^a^	4.31 (1.63) ^b^	4.42 (1.6) ^a^
	Hospital: Surgical Units		4.28 (1.6) ^a^	4.38 (1.5) ^a^	4.38 (1.64) ^b^	4.34 (1.58) ^a^
	Patient Management		4.30 (1.5) ^a^	4.51 (1.5) ^a^	4.39 (1.55) ^b^	4.4 (1.5) ^a^
	Mental Health		4.64 (1.08) ^a^	5.08 (0.8) ^b^	4.85 (1.05) ^b^	4.81 (0.97) ^b^
	Mother and Baby Units		4.32 (1.58) ^a^	4.41 (1.52) ^a^	4.42 (1.58) ^b^	4.38 (1.56) ^a^
Training	Nursing Diploma	0.00	4.44 (1.44) ^a^	4.69 (1.47) ^a^	4.54 (1.51) ^b^	4.59 (1.47) ^a^
	Doctorate		4.67 (1.29) ^a^	4.74 (1.28) ^b^	4.56 (1.49) ^b^	4.70 (1.35) ^b^
	Specialist via Internal Nursing Resident		4.47 (1.37) ^a^	4.67 (1.43) ^a^	4.63 (1.38) ^b^	4.59 (1.39) ^a^
	University Specialist		4.93 (1.66) ^b^	4.86 (1.71) ^b^	5.00 (1.6) ^b^	4.93 (1.65) ^b^
	Nursing Degree		4.89 (1.37) ^b^	5.12 (1.38) ^b^	4.99 (1.42) ^b^	5 (1.39) ^b^
	University Master’s		4.43 (1.41) ^a^	4.56 (1.54) ^a^	4.52 (1.46) ^b^	4.5 (1.47) ^a^

^a^ Medium Level; ^b^ High level; Abbreviation: DE Standard Deviation.

**Table 3:** Model adjustment and significance of the regression analysis.

Regression and Significance Coefficients					
R^2^ = 0.752 ^a^		Fisher’s F = 19.280 ^b^		*p* = 0.01 ^c^	
Variable	Coefficient		Degrees of Freedom	Fisher’s F	*p*-Value
Sex	0.156		1	12.348	0.00
Level of care	0.057		2	2.247	0.107
Type of unit	0.094		5	3.971	0.002
Training	0.14		4	12.875	0.00

^a^ Regression coefficient; ^b^ Fisher tests; ^c^ *p*-value.

## Data Availability

The datasets used and/or analyzed during the current study are available from the corresponding author on reasonable request.
